# Tetrahedral M_4_(μ_4_-O) Motifs Beyond Zn: Efficient One-Pot Synthesis of Oxido–Amidate Clusters *via* a Transmetalation/Hydrolysis Approach

**DOI:** 10.1021/acs.inorgchem.2c00456

**Published:** 2022-05-10

**Authors:** Piotr Krupiński, Michał Terlecki, Arkadiusz Kornowicz, Iwona Justyniak, Daniel Prochowicz, Jan van Leusen, Paul Kögerler, Janusz Lewiński

**Affiliations:** † Institute of Physical Chemistry, Polish Academy of Sciences, Kasprzaka 44/52, 01-224 Warsaw, Poland; ‡ Faculty of Chemistry, Warsaw University of Technology, Noakowsiego 3, 00-664 Warsaw, Poland; § Institute of Inorganic Chemistry, RWTH Aachen University, Landoltweg 1, 52074 Aachen, Germany

## Abstract

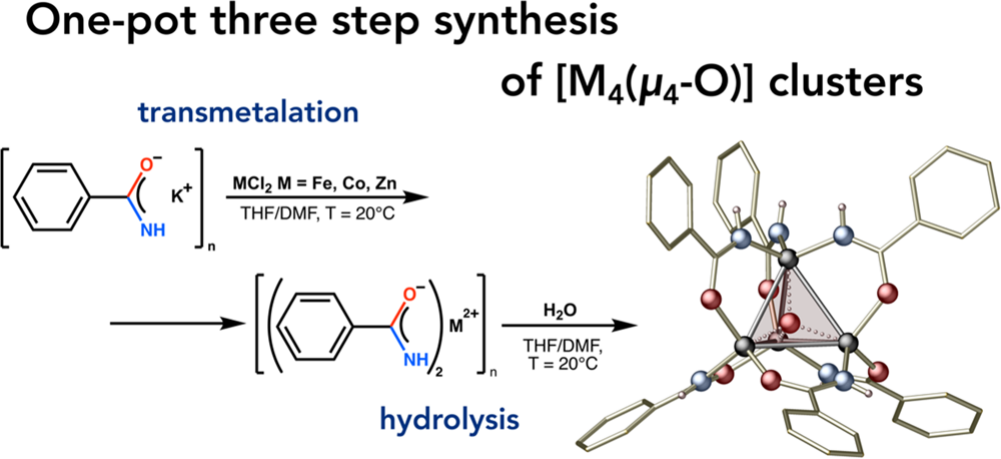

While zinc μ_4_-oxido-centered complexes are widely used as versatile precursors and building units of functional materials, the synthesis of their analogues based on other transition metals is highly underdeveloped. Herein, we present the first efficient systematic approach for the synthesis of homometallic [M_4_(μ_4_-O)L_6_]-type clusters incorporating divalent transition-metal centers, coated by bridging monoanionic organic ligands. As a proof of concept, we prepared a series of charge-neutral metal-oxido benzamidates, [M_4_(μ_4_-O) (NHCOPh)_6_] (M = Fe, Co, Zn), including iron(II) and cobalt(II) clusters not accessible before. The resulting complexes were characterized using elemental analysis, FTIR spectroscopy, magnetic measurements, and single-crystal X-ray diffraction. Detailed structural analysis showed interesting self-assembly of the tetrahedral clusters into 2D honeycomb-like supramolecular layers driven by hydrogen bonds in the proximal secondary coordination sphere. Moreover, we modeled the magnetic properties of new iron (II) and cobalt (II) clusters, which display a general tendency for antiferromagnetic coupling of the μ_4_-O/μ-benzamidate-bridged metal centers. The developed synthetic procedure is potentially easily extensible to other M(II)-oxido systems, which will likely pave the way to new oxido clusters with interesting optoelectronic and self-assembly properties and, as a result, will allow for the development of new functional materials not achievable before.

## Abstract 2

While zinc μ_4_-oxido-centered complexes are easily accessible building units of functional materials, the synthesis of their analogues based on other transition metals is highly underdeveloped. Herein, we present the first efficient systematic approach for the synthesis of [M_4_(μ_4_-O)]-type clusters incorporating divalent transition-metal centers. As prepared, a series of model metal-oxido benzamidates revealed interesting trends in their self-assembly and magnetic properties.

## Introduction

**Figure 1:**
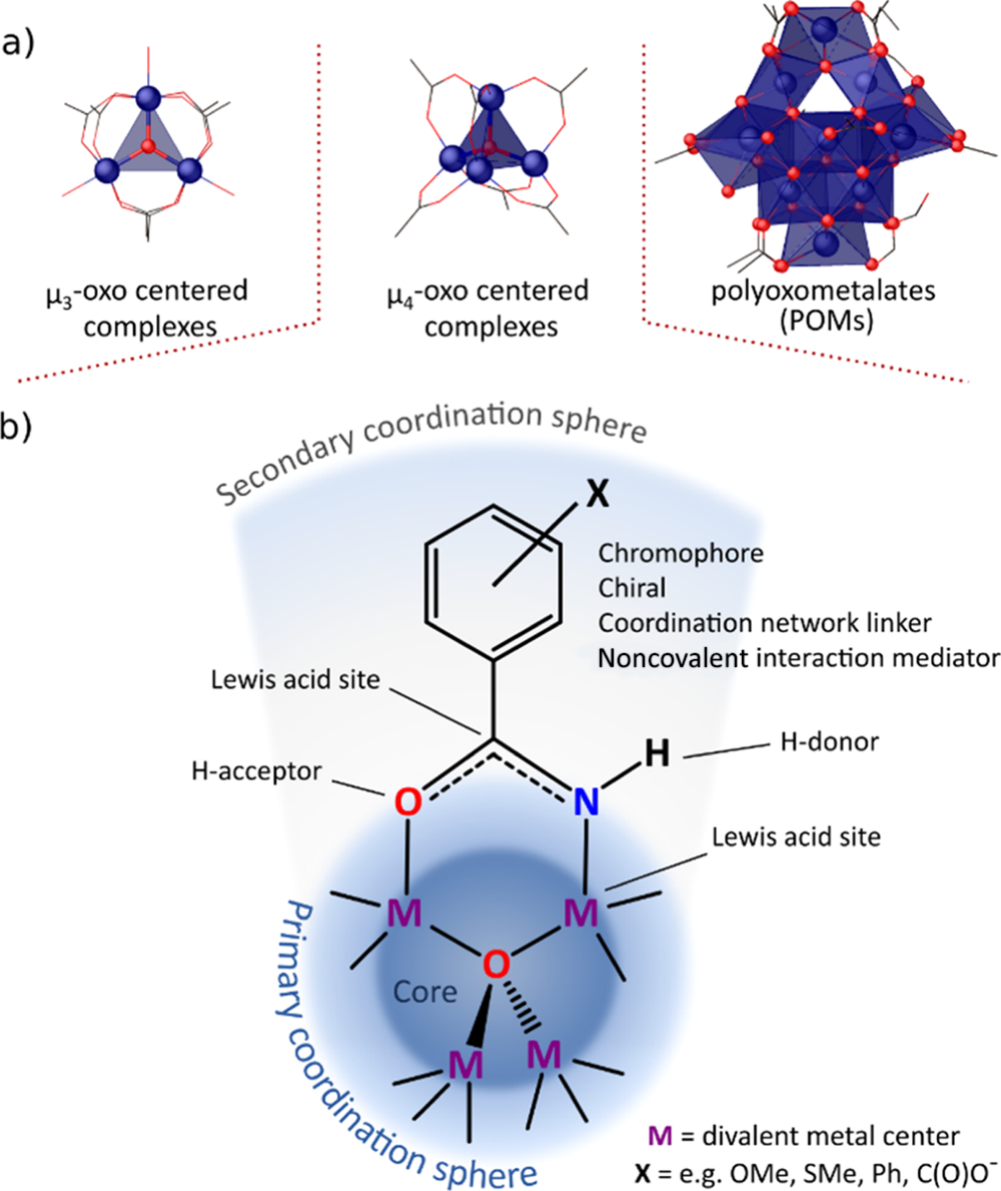
(a) Main families of metal-oxido compounds. (b) Multifaceted chemistry of tetrahedral μ_4_-oxido [M_4_(μ_4_-O)L_6_]-type complexes.

Molecular metal-oxido clusters have gained attention as versatile building units of a wide variety of functional materials based on both coordination^1,2^ and noncovalent supramolecular networks.^3,4^ The most prominent subsets of metal-oxido clusters include trinuclear μ_3_-oxido-centered complexes, tetranuclear μ_4_-oxido clusters, and multinuclear polyoxidometalates (POMs) (Figure 1a). Among them, clusters comprising a highly symmetrical tetrahedral [M_4_(μ_4_-O)]^6+^ core (M = divalent metal) stabilized by six monoanionic bidentate organic ligands are of particular interest due to their multifaceted chemistry arising from the presence of several spots of structural tailorability, including divalent metal centers in the tetrahedral core, the character of anchoring groups, and the organic backbone of ligands in the secondary coordination sphere (Figure 1b).^5,6^ Such diversity of structural features results in tuneable optoelectronic and coordination properties, which turns μ_4_-oxido compounds into suitable candidates for magnetic,^7^ luminescent,^8−10^ and catalytic^11−15^ materials. Moreover, [M_4_(μ_4_-O)L_6_]-type complexes can be regarded as discrete soluble intermediates between simple monomers and polymeric lattices of various hybrid organic–inorganic architectures. As such, they are widely used as model systems for studying properties of more complex systems including MOFs^16,17^ and hybrid metal oxide nanoparticles,^18,19^ as well as effective precursors of functional materials.^5,6,20−26^ Nevertheless, the development of materials containing the [M_4_(μ_4_-O)] structural motif beyond zinc clusters is hampered by the lack of facile access to this class of complexes comprising metal centers of desired optoelectronic, catalytic, or magnetic properties. For instance, access to homometallic isoreticular MOF-5 analogues based on metals other than zinc is still very limited. To the best of our knowledge, three Co(II) pyrazolate frameworks are the only examples of such materials obtained *via* direct synthesis from inorganic salts.^27−29^ Strikingly, this approach failed in the case of isoreticular frameworks based on dicarboxylate linkers. We also note that MOF-5(Co) and MOF-5(Be) were obtained from well-defined metal-oxido precursors using the controlled SBU approach,^20^ and cobalt (II) MOF-5 analogues were obtained by a post-synthetic transmetalation.^17,30^ However, the latter process was not effective for other metal ions.

**Figure 2:**
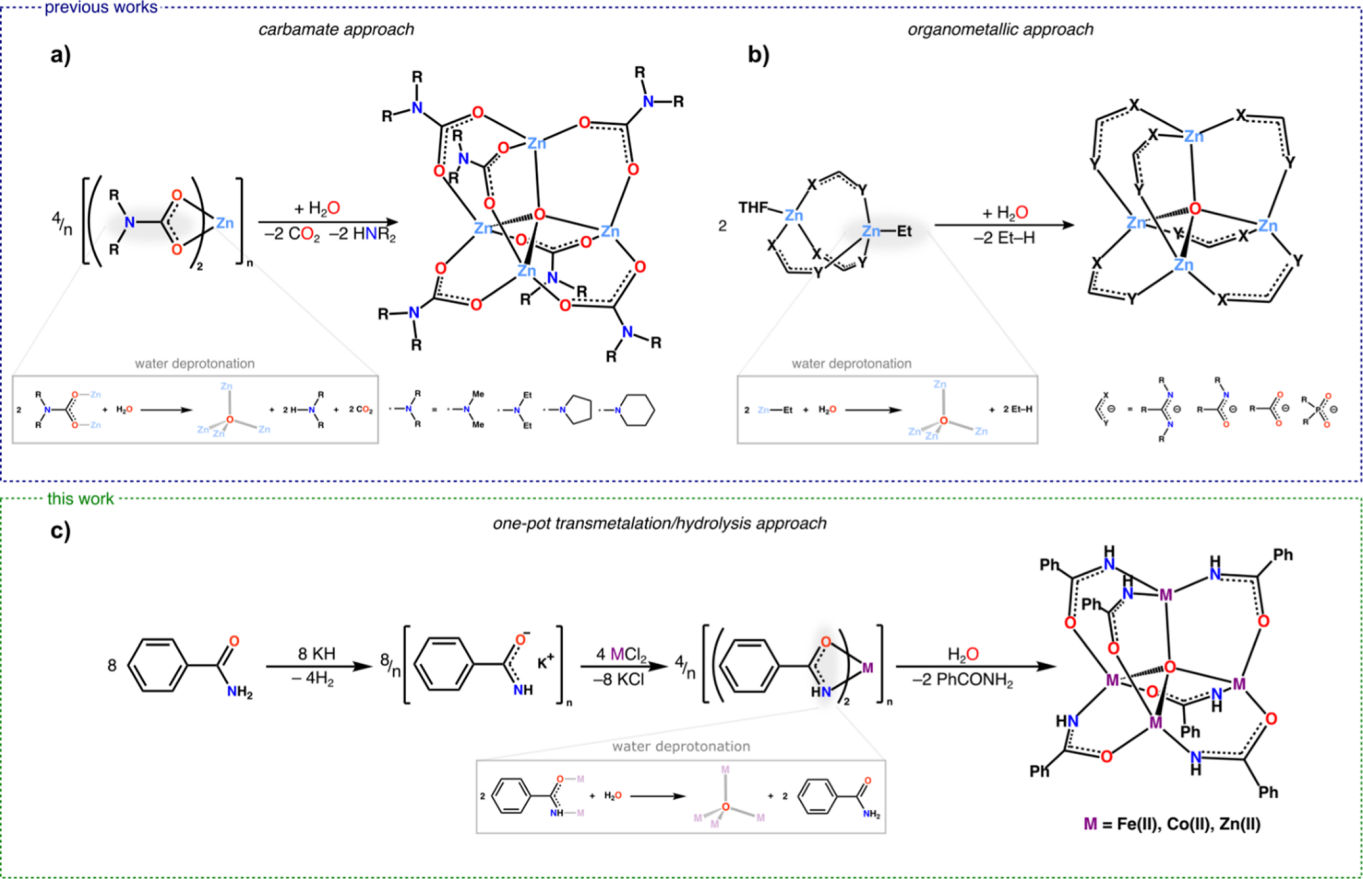
Controlled synthesis of metal μ_4_-oxido [M_4_(μ_4_-O)L_6_]-type clusters from well-defined precursors: (a) carbamate approach, (b) organometallic approach, and (c) one-pot transmetalation/hydrolysis approach.

To date, readily accessible zinc μ_4_-oxido clusters have been the subject of the most extensive investigations. For example, the most common zinc-oxido carboxylates are easily prepared by either thermal decomposition of zinc carboxylates *via* elimination of the corresponding anhydride^31^ or in alkaline solutions of zinc salts with water acting as a source of the O^2–^ ion.^10,32,33^ An increased control over the process of zinc-oxido carboxylates formation was achieved by using homoleptic zinc carbamates as well-defined precursors acting simultaneously as water deprotonation agents (Figure 2a).^34−37^ Another approach based on well-defined precursors utilizes diorganozinc species, which are reacted with the appropriate proligand, and then are exposed to dioxygen^38−40^ or undergo hydrolysis by addition of a stoichiometric amount of water (Figure 2b).^3,5,6,16,18,19,21,41−44^ In the hydrolytic transformation, the high Brønsted basicity of alkylzinc moieties is not only used to generate O^2–^ ions but also facilitates initial deprotonation of the proligand. This approach enabled the synthesis of μ_4_-oxido complexes incorporating a wide range of organic ligands, namely, carboxylates,^5,16,21^ phosphinates,^18,19,42^ amidates,^23^ amidinates,^6,26,41,43^ and guanidinates.^44^ Despite the wide scope of applied [Zn_4_(μ_4_-O)]^6+^ core coating ligands, this relatively universal approach for Zn–oxido complexes is essentially non-transferable to the related transition-metal systems.

In contrast to zinc–oxido complexes, tetrahedral open-shell transition metal–oxido complexes were mostly obtained serendipitously, seldom by design.^45^ For example, Mn(II), Fe(II), and Co(II) μ_4_-oxido clusters stabilized by *N*,*N*′-bidentate ligands were isolated from reactions of inorganic metal salts with lithiated ligands.^46−48^ In all cases, contamination by atmospheric oxygen or moisture was indicated as the source of the O^2–^ ions. Similarly, a 3,5-dimethylpyrazolate cobalt-oxido cluster was obtained unexpectedly during attempts to synthesize the mixed cobalt/zinc [Co_1/3_Zn_2/3_(H*dmpz*)_2_]_*x*_ (H*dmpz* = 3,5-dimethylpyrazole) complex.^49^ Furthermore, to the best of our knowledge, the carbamate cluster [Co_4_(μ_4_-O) (OOCNC_9_H_18_)_6_], obtained *via* the insertion of a TEMPO radical into Co_2_(CO)_8_^7,50^ is the only [M_4_(μ_4_-O)L_6_] system characterized magnetically. Therefore, we believe that the application potential of tetrahedral oxido-centered clusters as functional materials cannot be fully revealed without a reliable synthesis method applicable to metal centers of various characters.

Herein, we present a novel one-pot transmetalation/hydrolysis procedure for the synthesis of homometallic oxido clusters incorporating various divalent metal centers (Figure 2c). The efficiency of the developed approach was demonstrated by the preparation of an amide-stabilized zinc-oxido complex [Zn_4_(μ_4_-O)(NHCOPh)_6_] (**1-Zn**) and its hitherto inaccessible iron (II) and cobalt (II) analogues (**1-Fe** and **1-Co**). The structural characterization of **1-Fe**, **1-Co**, and **1-Zn** revealed that they all exhibit similar self-assembly properties resulting in a honeycomb-like supramolecular motif. Furthermore, magnetic characterization of **1-Fe** and **1-Co** shows strong antiferromagnetic interactions between the metal centers within the [M_4_(μ_4_-O)] core likely mediated by the tetrahedral O^2–^ bridges.

## Experimental Section

### General Considerations

All manipulations were conducted under a dry, oxygen-free argon atmosphere either using standard Schlenk techniques or in a glovebox. All reagents were purchased from commercial vendors: benzamide (Sigma), KH (Sigma), cobalt (II) chloride (ABCR), iron (II) chloride (ABCR), zinc chloride and used as received. Solvents were purified using an MBraun SPS-5 system.

### General Procedure for the Synthesis of **1-Fe**, **1-Co**, and **1-Zn**

Equimolar amounts of benzamide (0.969 g, 8.00 mmol) and KH (0.320 g, 8 mmol) were placed in a Schlenk flask cooled to 0 °C and dispersed in THF (30 mL). After a few minutes, the reaction mixture was allowed to gradually warm to room temperature and stirred for 24 h. Then, FeCl_2_ (0.507 g, 4 mmol), CoCl_2_ (0.519 g, 4 mmol), or ZnCl_2_ (0.545 g, 4 mmol) was added. After another 8 h of stirring, 18 μL (1 mmol) of Millipore water and 10 mL of dimethylformamide (DMF) were added and the reaction mixture was stirred for another 24 h. The product was isolated as red-brown (**1-Fe**), dark blue (**1-Co**), or colorless (**1-Zn**) crystals after filtration and crystallization in the presence of hexane vapor at room temperature (yield = 71%, 755 mg (**1-Fe**); 79%, 859 mg (**1-Co**); 75%, 830 mg (**1-Zn**)).

### Characterization

FTIR spectra were measured with a Bruker Tensor II spectrometer using the ATR technique. Elemental analyses were performed using a UNICUBE elemental analyzer (Elementar Analysensysteme GmbH). Powder XRD data were collected on a PANalytical Empyrean diffractometer with Ni-filtered Cu Kα radiation (λ = 1.5406 Å) or Bruker D8 Advance diffractometer with V-filtered Cr Kα radiation (λ = 2.2897 Å). The sample was spread on a surface of a silicon plate fixed to the sample holder and sealed by a Capton tape. Diffraction patterns were collected by scanning with a step of 0.02°.

**1-Fe**: IR (ATR): ν/cm^–1^ 3347 (w), 3065 (vw), 2951 (vw), 2862 (vw), 1667 (m), 1599 (s), 1540(vs), 1508 (w), 1438 (vs), 1220 (s), 1117 (m), 1030 (w), 933 (w), 699 (vs), 668 (s), 472 (vs). Elemental analysis: calcd (%) for **1-Fe**·0.84 THF·0.58 DMF (C_47.1_H_46.8_N_6.6_O_8.4_Fe_4_) (1063.13): C, 53.21; H, 4.44; N, 8.67. Found (%): C, 53.33; H, 4.32; N, 8.77.

**1-Co**: IR (ATR): ν/cm^–1^ 3348 (w), 3068 (vw), 2973 (vw), 2863 (vw), 1674 (m), 1599 (s), 1555(vs), 1506 (w), 1454 (vs), 1388 (w), 1234 (s), 1133 (m), 1030 (w), 701 (vs), 670 (s), 489 (vs). Elemental analysis: calcd (%) for **1-Co**·0.93 THF·0.59 DMF (C_47.5_H_47.5_N_6.6_O_8.5_Co_4_) (1082.70): C, 52.68; H, 4.43; N, 8.53. Found (%): C, 52.91; H, 4.19; N 8.72.

**1-Zn**: ^1^H NMR (300 MHz, CDCl_3_): δ [ppm] 7.90–7.73 (m, 12H, Ph), 7.46–7.32 (m, 18H, Ph), 6.36 (s, 1.2H, NH), 6.24 (s, 2.4H, NH), 6.15 (s, 1.2H, NH); ^13^C NMR (75 MHz, CDCl_3_): δ [ppm] 177.12 (NCO), 137.03 (^IV^C_Ar_), 130.55 (C_Ar_), 128.14 (C_Ar_), 127.36 (C_Ar_); IR (ATR): ν/cm^–1^ 3361 (w), 3063 (vw), 2976 (vw), 2863 (vw), 1672 (m), 1599 (s), 1557 (s), 1501 (m), 1449 (vs), 1388 (w), 1233 (s), 1131 (m), 1028 (w), 697 (vs), 674 (s), 488 (s). Elemental analysis: calcd (%) for **1-Zn**·0.68 THF·0.75 DMF (C_47_H_47_N_6.75_O_8.43_Zn_4_) (1102.16): C, 51.19; H, 4.27; N, 8.58. Found (%): C, 51.22; H, 4.22; N, 8.61.

### X-ray Structure Determination

The data were collected at 100(2) K on a Nonius Kappa CCD diffractometer^51^ using graphite monochromated Mo Kα radiation (λ = 0.71073 Å). The crystal was mounted in a nylon loop in a drop of silicon oil to prevent the possibility of decay of the crystal during data collection. The unit cell parameters were determined from ten frames and then refined on all data. The data were processed with DENZO and SCALEPACK (HKL2000 package).^52^ The structure was solved by direct methods using the SHELXS-97 program and was refined by full matrix least-squares on F^2^ using the program SHELXL-97.^53^ All non-hydrogen atoms were refined with anisotropic displacement parameters. The hydrogen atoms were introduced at geometrically idealized coordinates with a fixed isotropic displacement parameter equal to 1.5 (methyl groups) times the value of the equivalent isotropic displacement parameter of the parent carbon. Crystallographic data (excluding structure factors) for the structure reported in this paper have been deposited with the Cambridge Crystallographic Data Centre as a supplementary publication. Copies of the data can be obtained free of charge on application to CCDC, 12 Union Road, Cambridge CB21EZ, UK (fax: (+44)1223-336-033; e-mail: deposit@ccdc.cam.ac.uk). CCDC: 2122831 (**1-Fe**); 2122830 (**1-Co**); 2122832 (**1-Zn**).

### Magnetic Measurements

Magnetic properties were determined using a Quantum Design MPMS–5XL SQUID magnetometer for direct current (dc) and alternating current (ac) measurements. Microcrystalline samples of **1-Fe** and **1-Co** were compacted and immobilized into cylindrical PTFE sample holders. Experimental dc data were recorded at 0.1 T in the temperature range 2.0–290 K and at 2.0 K in the field range 0.1–5.0 T. Experimental ac data were collected at a zero static bias field in the temperature range 2.0–50 K and frequency range 3–1000 Hz using an amplitude of *B*_ac_ = 3 G. However, no relevant out-of-phase signals were detected for either compound. All data were corrected for the diamagnetic contributions of the sample holders and the compound (χ_m,dia_/10^–4^ cm^3^ mol^–1^ = −5.37 (**1-Fe**), −5.31 (**1-Co**)).

## Results and Discussion

### Synthesis of [M_4_(μ_4_-O)(NHC(O)Ph)_6_] (M = Fe, Co, Zn) Complexes

Metal μ_4_–oxido complexes were prepared using a new synthetic procedure that involves three main steps: (i) generation of a potassium salt from the selected proligand L-H, (ii) transmetalation reaction with M(II) salt, and (iii) stoichiometric hydrolysis of the received homoleptic M(II) complex (Figure 1c). Benzamide-coated clusters were selected as model systems due to their close resemblance to the SBUs of the archetypal MOFs. To demonstrate the applicability of the proposed methodology, we first performed the synthesis of the zinc–oxido complex (**1-Zn**). Subsequently, we prepared the Co(II)- and Fe(II)-based μ_4_–oxido complexes (**1-Co** and **1-Fe**), as examples of the elusive homologues based on magnetically active and redox-active elements.

In a typical procedure, equimolar amounts of KH and benzamide were placed in a Schlenk flask and dispersed in THF. The reaction mixture was stirred overnight, and then, a 0.5 molar equivalent of MCl_2_ (M = Fe, Co, Zn) was added. After another 12 h, the obtained red-brown (Fe), deep blue (Co), or white (Zn) suspensions were hydrolyzed using the 1:4 H_2_O/M molar ratio. To increase the solubility of the resulting metal–oxido complexes, DMF was added to the reaction mixture, and the solution was stirred overnight. Then, the KCl slurry was removed by filtration, and the respective product was isolated as well-shaped red-brown (Fe), deep blue (Co), or colorless (Zn) crystals (Figure S8) by slow diffusion of hexane vapor into the parent solution (for systematic characterization of the resulting complexes *vide infra*).

A key aspect of the developed process is the highly efficient metathesis transmetalation driven by the low solubility of KCl in the employed organic solvent. The use of a potassium salt also allows to effectively eliminate the incorporation of alkali ions into the final product, which was reported to occur in the case of Li- or Zn-based salts used in transmetalation processes.^22,39,43,54−56^ Advantageously, potassium salts of highly basic ligands like amides can be easily generated *in situ* by the reaction of a selected proligand L-H with KH, where the only side product is H_2_, which finally enables a one-pot synthesis. Furthermore, the utilization of ligands with sufficiently high basicity is a crucial factor for ensuring proper reactivity of a homoleptic ML_2_ complex, which acts as both (i) the source of M(II) ions and stabilizing ligands, as well as (ii) the water-deprotonating agent. Overall, the transmetalation/hydrolysis process leads to the desired product [M_4_(μ_4_-O)L_6_] in high purity, with the generation of three easy-to-separate side products: (i) gaseous H_2_ leaving the reaction system, (ii) KCl precipitate that can be removed by filtration, and (iii) the starting proligand, which can be separated from the product by crystallization and reused. Due to the above-mentioned qualities, the developed methodology is potentially easily applicable to other μ_4_-O systems based on various M(II) metal centers and a wide range of organic ligands with high basicity such as amidinate, ureate, or guanidinate anions.

### Structural Characterization

Complexes **1-Fe**, **1-Co**, and **1-Zn** were characterized spectroscopically and by single-crystal X-ray diffraction and elemental analysis. Compound **1-Zn** was previously obtained *via* the organometallic synthesis,^23^ and the current characteristics (for details, see Experimental Section and Supporting Information) are consistent with the previous results. The FTIR spectra of **1-Fe** and **1-Co** are analogous to **1-Zn** indicating the presence of monoanionic benzamide ligands along with the [M_4_(μ_4_-O)] core characterized by the M-O bands observed at 477, 487, and 488 cm^–1^ for iron, cobalt, and zinc cluster, respectively (Figure S9). Analysis of the crystal structures indicates that **1-Fe**, **1-Co**, and **1-Zn** are isostructural and crystalize in the *P*3̅*c*1 space group as solvates with THF and DMF. The elemental (see Experimental Section) and ^1^H NMR analysis (Figure S10) indicates the **1**:THF/DMF stoichiometry as approximately 1:0.75:0.75; however, the solvent content can vary in bulk samples.

**Figure 3:**
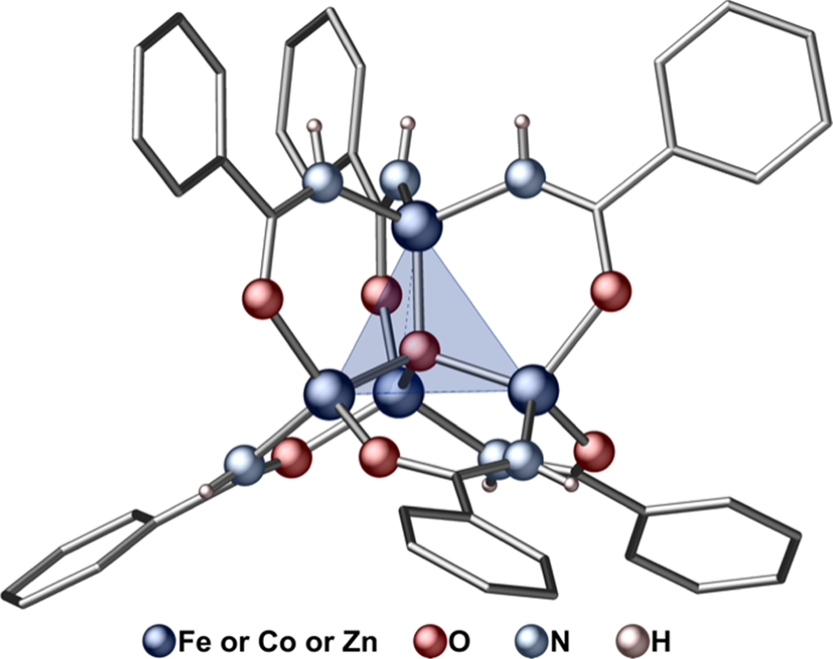
Molecular structure of the isostructural complexes **1-Fe**, **1-Co**, and **1-Zn**; detailed information regarding the structures are provided in the Supporting Information.

The molecular structure of the complexes **1-Fe**, **1-Co**, and **1-Zn** comprises a tetrahedral [M_4_(μ_4_-O)] core coated by six μ_2_ monoanionic benzamidate ligands (Figure 3). The M–O bonds to the central O^2–^ ion are in the range 1.973–1.974, 1.948–1.955, and 1.941–1.961 Å for **1-Fe**, **1-Co**, and **1-Zn**, respectively. These values are in line with the M–O bond lengths observed in previously reported iron (II) (1.952–1.955 Å),^46^ cobalt (II) (1.928–1.956 Å),^50^ and zinc (II) (1.935–1.966 Å)^5,6^ tetrahedral oxido clusters. In turn, the M–O and M–N metal-to-amidate bond lengths (**1-Fe**: 1.972–2.023, **1-Co**: 1.941–1.996, **1-Zn**: 1.958–2.005 Å) are longer than typical M–O (Fe–O: 1.936–1.978 Å,^57^ Co–O: 1.929–1.950 Å,^50^ Zn–O: 1.922–1.962 Å^5^) but shorter than typical M–N (Fe–N: 2.044–2.070 Å,^46^ Co–N: 2.000–2.059 Å,^46^ Zn–N: 1.997–2.035 Å^6,46^) bond lengths in known oxido–metal clusters. This observation indicates a disorder in the arrangement of N and O atoms in the crystal structures of **1-Fe**, **1-Co**, and **1-Zn**, which suggests the occurrence of the coordination position isomerism in the benzamidate anions. Specifically, each of them may act either as an (O,N)- or (N,O)-bidentate ligand, which overall gives four isomers bearing metal centers with varying coordination environments: M^(NNN)^M^(OON)^M^(OON)^M^(OON)^, M^(NNN)^M^(OOO)^M^(OON)^M^(NNO)^, M^(NNO)^M^(OOO)^M^(NNO)^M^(NNO)^, and M^(NNO)^M^(NOO)^M^(NNO)^M^(NOO)^ (Figure S5). Furthermore, each of these isomers is chiral, which adds up to four pairs of possible enantiomers occupying the same position in the crystal lattice. This coordination position isomerism introduces differences in environments around N-bonded protons, which explains the observed respective signal splitting in the ^1^H NMR spectrum (Figure S10, for details see Supporting Information).

**Figure 4:**
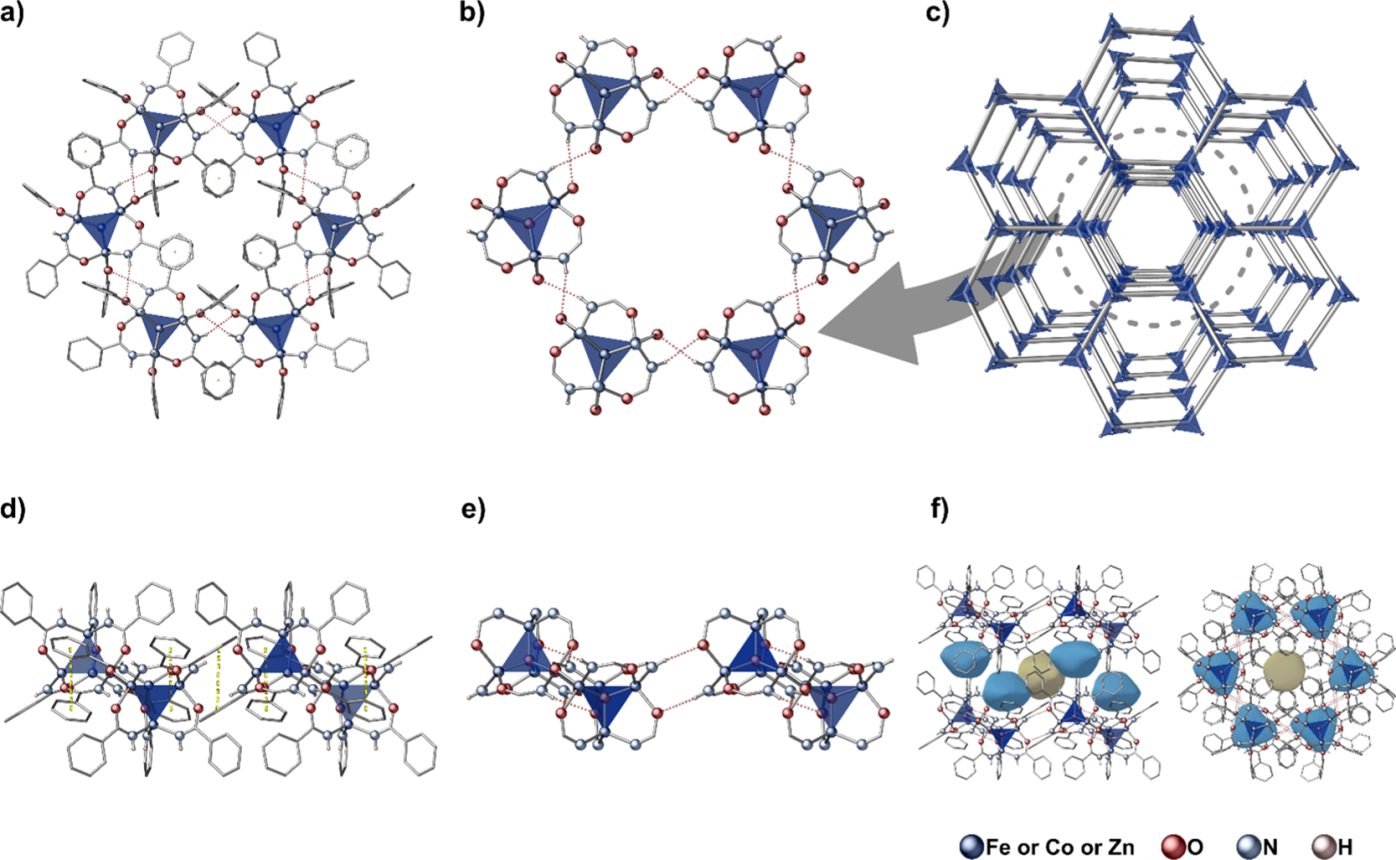
Crystal structure motifs of **1-Fe**, **1-Co**, and **1-Zn**: (a,d) hexagonal assembly of oxido clusters driven by noncovalent interactions in the secondary coordination sphere; (b,e) system of intermolecular hydrogen bonds between oxido clusters; (c) schematic representation of the honeycomb-like supramolecular structure; (f) solvent accessible surfaces of the two types (blue and yellow) of closed voids between 2D supramolecular honeycomb-like layers.

Very recently, we have demonstrated that the N-bonded hydrogen atoms in the proximal secondary coordination sphere provide efficient H-donor sites for hydrogen bonds which influence the self-assembly of zinc-oxido clusters.^6^ In this context, we showed that complex **1-Zn**, comprising both O and NH groups in the anchoring group of stabilizing ligand, self-assembles into 2D supramolecular honeycomb-like layers *via* pairs of complementary intermolecular NH···O hydrogen bonds (the N–H···O distance is 2.760 Å) supported by π–π interactions in the distal secondary coordination sphere (Figures 4 and S4). The new complexes **1-Fe** and **1-Co** exhibit similar self-assembly properties with the corresponding intermolecular O···HN distances of 2.662 and 2.783 Å, respectively (Table S10), as well as analogous packing of molecules in the crystal structure. The 2D honeycomb supramolecular layers are AA-type stacked, as a consequence forming two types of closed voids: one in the environment of two [M_4_(μ_4_-O)] cores and another one within the space limited by aromatic rings of 12 neighboring clusters (Figure 4f). These voids are filled by disordered THF or DMF solvent molecules (1.5 solvent molecules per oxido cluster). THF molecules enclosed in the voids of the former type are partially oriented due to the formation of O···HN hydrogen bonds involving the amidate NH groups.

### Magnetic Characterization

**Figure 5:**
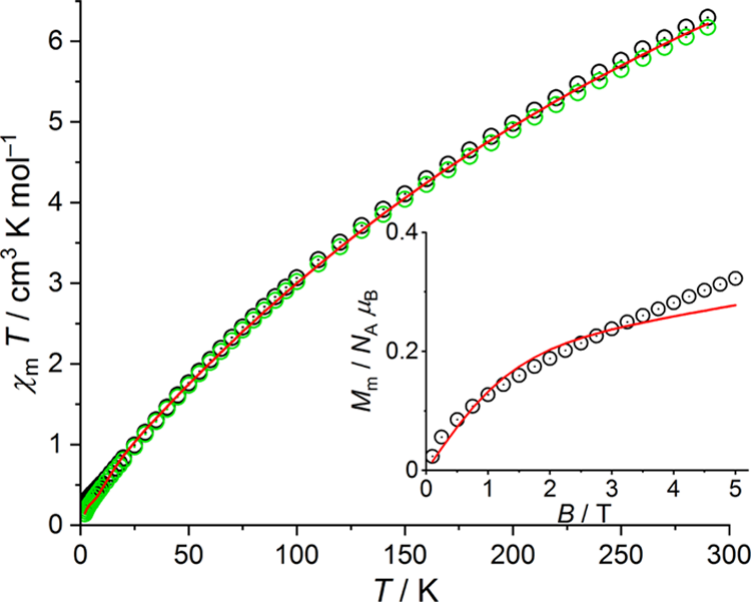
Temperature dependence of χ_m_*T* at 0.1 (black) and 1.0 T (green circles) of **1-Fe**; inset: molar magnetization *M*_m_ vs magnetic field *B* at 2.0 K; red lines represent the least-squares fit (above 10 K) to the effective spin model described in the text.

**Figure 6:**
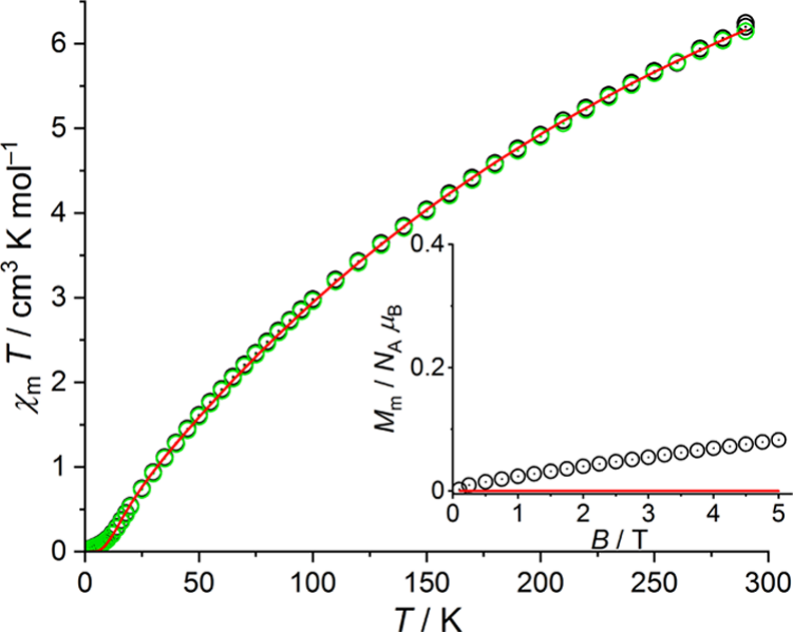
Temperature dependence of χ_m_*T* at 0.1 (black) and 1.0 T (green circles) of **1-Co**; inset: molar magnetization *M*_m_ vs magnetic field *B* at 2.0 K; red lines represent the least-squares fit to the effective spin model described in the text.

Comprehensive characterization of physicochemical properties of molecular building blocks is crucial for designing new functional supramolecular assemblies. Notably, the exploration of regular [M_4_(μ_4_-O)L_6_]-type magnetic systems with uniform exchange pathways (i.e., coupling patterns) between all spin centers has been impeded by the inaccessibility of well-defined model complexes. To the best of our knowledge, the carbamate cluster [Co_4_(μ_4_-O)(OOCNC_9_H_18_)_6_] is the only system of this type characterized magnetically, albeit on a purely phenomenological level indicating antiferromagnetic interaction between the cobalt ions.^7^ However, tetranuclear Cu(II) cluster [Cu_4_(μ_4_-O)(OOCCF_3_)_6_(q)_4_] (q = quinoline) can be described as a farther-related analogue of such regular systems, based on pentacoordinate, trigonal bipyramidal metal centers.^58^ Its magnetic properties were extensively studied experimentally and theoretically indicating exceptionally ferromagnetic interactions within the [M_4_(μ_4_-O)] core. All other works on the magnetic properties of μ_4_-oxido-centered complexes involve more distorted systems with less uniform ligand spheres, commonly including monoatomic chalcogenide or alkoxide bridges prone to the mediation of strong antiferromagnetic interactions.^59−62^ Taking this into account, we performed magnetic characterization of the new complexes **1-Fe** and **1-Co**. The magnetic data indicates predominantly antiferromagnetic exchange interactions between four M(II) centers within the [M_4_(μ_4_-O)] core for both Co(II) and Fe(II) systems. This is reflected by the shape of the χ_m_*T* versus *T* plot, the rather low value of χ_m_*T* at 290 K, and the almost vanishing values of χ_m_*T* and *M*_m_ at 2.0 K representing an effective total spin value of *S*_total_ = 0 (Figures 5 and 6, see Supporting Information for a detailed discussion). These observations are consistent with the previous magnetic study for [Co_4_(μ_4_-O)(OOCNC_9_H_18_)_6_], which leads to the general conclusion that the tetrahedral oxido-bridges in [M_4_(μ_4_-O)] systems tend to mediate strong antiferromagnetic coupling. This phenomenon is often linked to the high M–O–M bond angles,^63,64^ which in **1-Fe** and **1-Co** fall in the range 106.3–112.4°. We also analyzed the magnetic properties of **1-Fe** and **1-Co** using an effective isotropic spin model, which here represents a reasonable approximation since tetrahedrally coordinated 3d^6^ and 3d^7^ centers are characterized by ^5^E and ^4^A_2_ ground terms, respectively. In contrast, due to the larger spin–orbit coupling of both centers, the ground terms are usually split, leading to temperature-dependent behavior of χ_m_*T* below 30 K. However, this feature can be neglected for **1-Co** as a relevant splitting of the A_2_ ground term requires a strong distortion from the tetrahedral ligand field, which is not the case for the molecular structure of **1-Co**. For **1-Fe**; however, the quintet E term is significantly split in any case. Therefore, the χ_m_*T* versus *T* data for both compounds were simultaneously fitted at both 0.1 and 1.0 T, restricted to the temperature interval of 10–290 K and subsequently the values at lower temperatures were calculated. Based on the single-crystal X-ray structures, we model the M_4_ cluster as a trigonal pyramid, with the apex defined by threefold N-coordinated metal, since the distance from the central O here slightly differs from the other centers (as shown in Figure 3). Accordingly, we define the spin connectivity by two different exchange parameters: *J*_b_, between the metal centers of the base (denoted by indices 2–4) and *J*_t_, between a metal center of the base and the apical (top) metal center (index 1). Thus, the corresponding effective Hamiltonian is
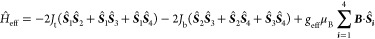
1

The data of **1-Fe** indicate a small level of paramagnetic impurities, which was accounted for in the analysis of the data. For **1-Co**, we neglected such contributions, since the measurements point to a negligible level, that is, within the error margin of the fitting model. For **1-Fe**, consisting of effective *S* = 2 centers, the least-squares fit yields *J*_t_ = (−18.6 ± 0.1) cm^–1^, *J*_b_ = (−15.7 ± 0.2) cm^–1^, and *g*_eff_ = 2.21 ± 0.02, and a paramagnetic impurity level corresponding to 0.0964 Fe^II^ centers per formula unit. For **1-Co** (effective *S* = 3/2 centers), we find *J*_t_ = (−24.5 ± 0.1) cm^–1^, *J*_b_ = (−19.6 ± 0.1) cm^–1^, and *g*_eff_ = 2.56 ± 0.02. In both cases, the exchange interactions are antiferromagnetic, and the ground state of the respective compound can be characterized by an effective total spin *S*_total_ of 0. For each **1-Fe** and **1-Co**, the obtained two *J* values correspond to the geometric differences, in particular the M–(μ_4_-O)–M angles. Furthermore, the *J* values for the Fe^II^ and Co^II^ systems are very comparable when considering the different spin quantum numbers that enter the effective spin Hamiltonian as *S*^2^. The effective *g* factors are fully in line with tetrahedrally coordinated Fe^II^ and Co^II^ centers.

## Conclusions

We have developed a new efficient one-pot synthetic approach for tetrahedral μ_4_–oxido complexes of various divalent metal centers stabilized by monoanionic bridging O,N-ligands. The new synthetic procedure relies on simple stoichiometric hydrolysis of the homoleptic [ML_2_] complex generated *in situ* in a transmetalation reaction. To prove the usefulness of the developed approach, we have synthesized and characterized a series of M(II) (M = Fe, Co, Zn) oxido benzamidates. All complexes exhibit analogous honeycomb-like supramolecular structures in their crystal lattices, which indicates that their self-assembly properties are dominated by cooperative noncovalent interactions in the secondary coordination sphere and likely unaffected by the specific character of the metal center. Moreover, we have characterized the magnetic properties of the new Fe(II) and Co(II) complexes, utilizing effective spin models that underscore a general tendency for antiferromagnetic coupling of μ_4_-bridged metal centers in [M_4_(μ_4_-O)]-type systems.

The reported procedure is potentially easily extensible to other M(II)–oxido systems stabilized by a vast array of organic ligands. These findings will likely pave the way to new oxido clusters with interesting optoelectronic and novel molecular building units and, as a result, will allow for the development of new, as of yet elusive functional materials.

## Supporting Information Available

SC-XRD data, additional description of molecular structures and magnetic properties, PXRD patterns, and FTIR and NMR spectra (PDF)

## Accession Codes

CCDC 2122830–2122832 contain the supplementary crystallographic data for this paper. These data can be obtained free of charge via www.ccdc.cam.ac.uk/data_request/cif, or by emailing data_request@ccdc.cam.ac.uk, or by contacting The Cambridge Crystallographic Data Centre, 12 Union Road, Cambridge CB2 1EZ, UK; fax: +44 1223 336033.
