# Supplementary material for: Tetrahedral M4(μ4-O) Motifs Beyond Zn: Efficient One-Pot Synthesis of Oxido–Amidate Clusters via a Transmetalation/Hydrolysis Approach
Source: Inorg Chem. 2022 May 10;61(20):7869–77. doi: 10.1021/acs.inorgchem.2c00456 (PMC9131453; doi:10.1021/acs.inorgchem.2c00456)
Supplement: Supplementary file 1 — ic2c00456_si_001.pdf [file ic2c00456_si_001.pdf]

Supporting Information

Tetrahedral $M_4(\mu_4\text{-}O)$ Motifs Beyond Zn: Efficient One-Pot Synthesis of Oxido-Amidate Clusters *via* a Transmetalation/Hydrolysis Approach

Piotr Krupiński[†,§] Michał Terlecki,[‡,§] Arkadiusz Kornowicz,[,†] Iwona Justyniak,[†] Daniel Prochowicz,[†] Jan van Leusen,[⫽] Paul Kögerler,[⫽] and Janusz Lewiński[*,†,‡]*

[†]*Institute of Physical Chemistry, Polish Academy of Sciences, Kasprzaka 44/52, 01-224 Warsaw, Poland*
[‡]*Faculty of Chemistry, Warsaw University of Technology, Noakowsiego 3, 00-664 Warsaw, Poland*
[⫽]*Institute of Inorganic Chemistry, RWTH Aachen University, Landoltweg 1, 52074 Aachen, Germany*

*lewin@ch.pw.edu.pl

Contents

Crystallographic Data S2
Isomerism........ S11
Powder X-Ray Diffraction........ S12
FTIR Spectra S14
NMR Spectra S15
Magnetic Studies........ S17

Crystallographic Data

Table S1. Crystal data and structure refinement for **1-Fe**

Empirical formula	$C_{46}H_{44}Fe_4N_6O_8$	
Formula weight	1032.27 g mol^{-1}	
Temperature	100(2) K	
Wavelength	0.71073 Å	
Crystal system	Trigonal	
Space group	P -3 c 1	
Unit cell dimensions	a = 14.8151(6) Å	α = 90°
	b = 14.8151(6) Å	β = 90°
	c = 24.5407(8) Å	γ = 120°
Volume	4664.7(4) $Å^3$	
Z	4	
Density (calculated)	1.470 Mg/m^3	
Absorption coefficient	1.277 mm^{-1}	
F(000)	2120	
Crystal size	0.16 x 0.11 x 0.07 mm^3	
Theta range for data collection	3.176 to 26.487°	
Index ranges	$-18 \le h \le 14$, $-11 \le k \le 18$, $-30 \le l \le 23$	
Reflections collected	3231	
Independent reflections	3231 [R(int) = 0.0603]	
Completeness to theta = 25.242°	99.8 %	
Refinement method	Full-matrix least-squares on F^2	
Data / restraints / parameters	3231 / 9 / 198	
Goodness-of-fit on F^2	1.069	
Final R indices [$I > 2\sigma(I)$]	R1 = 0.0893, wR2 = 0.2027	
R indices (all data)	R1 = 0.1150, wR2 = 0.2174	
Largest diff. peak and hole	0.812 and –0.717 e $Å^{-3}$	

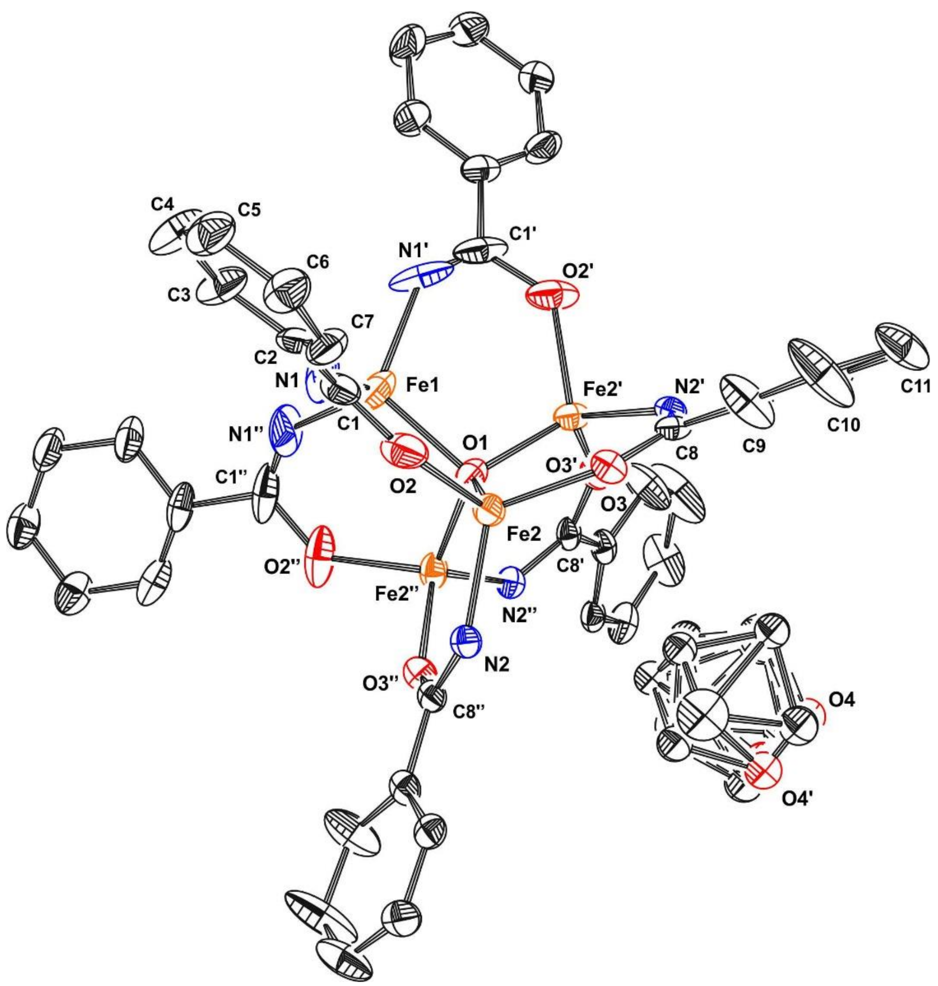

Figure S1. ORTEP diagram of molecular structure of **1-Fe** with thermal ellipsoids set at 30% probability; hydrogen atoms have been omitted for clarity. Symmetry transformations used to generate equivalent atoms: (–*x*+*y*+1, –*x*+1, *z*), (–*y*+1, *x*–*y*, *z*)

Table S2. Selected bond lengths [Å] and angles [°] for **1-Fe**

Fe1 – O1	1.973(7)	Fe1 – O1 – Fe2	106.3(2)
Fe1 – N1	2.023(10)	Fe2 – O1 – Fe2’	112.4(2)
Fe2 – O1	1.974(2)	O1 – Fe1 – N1	110.0(3)
Fe2 – O2	2.019(6)	O1 – Fe2 – O2	115.3(3)
Fe2 – O3	1.972(5)	O1 – Fe2 – O3	107.19(16)
Fe2 – N2	2.017(5)	O1 – Fe2 – N2	110.19(18)
C1 – O2	1.340(13)	Fe1 –N1 – C1	138.6(9)
C1 – N1	1.175(16)	Fe2 – O2 – C1	127.8(8)
C8 – O3	1.263(8)	Fe2 – O3 – C8	137.7(4)
C8 – N2’	1.310(8)	Fe2 – N2 – C8	129.6(5)

Table S3. Geometry of selected intermolecular hydrogen bonds in **1-Fe** (based on Platon)[1]

contact	H···A	D···A	D–H···A	symmetry
N1–H1···O4	2.702	3.459(2)	144.97	–*x*+*y*+1, +*y*, +*z*–1/2
N2–H2···O2	2.662	3.502(8)	159.79	*y*, +*x*, –*z*+1/2

Table S4. Crystal data and structure refinement for **1-Co**

Empirical formula	$C_{96}H_{96}Co_8N_{12}O_{17}$	
Formula weight	2161.28 g mol^{-1}	
Temperature	100(2) K	
Wavelength	0.71073 Å	
Crystal system	Trigonal	
Space group	P -3 c 1	
Unit cell dimensions	a = 14.7159(3) Å	α = 90°
	b = 14.7159(3) Å	β = 90°
	c = 24.7194(4) Å	γ = 120°
Volume	4636.0(2) Å^3	
Z	2	
Density (calculated)	1.548 Mg/m^3	
Absorption coefficient	1.468 mm^{-1}	
F(000)	2216	
Crystal size	0.18 x 0.11 x 0.05 mm^3	
Theta range for data collection	2.888 to 26.498°	
Index ranges	$-18 \le h \le 18$, $-18 \le k \le 18$, $-31 \le l \le 29$	
Reflections collected	43863	
Independent reflections	3215 [R(int) = 0.0483]	
Completeness to theta = 25.242°	99.8 %	
Refinement method	Full-matrix least-squares on F^2	
Data / restraints / parameters	3215 / 18 / 218	
Goodness-of-fit on F^2	1.183	
Final R indices [I>2σ(I)]	R1 = 0.0791, wR2 = 0.1532	
R indices (all data)	R1 = 0.0969, wR2 = 0.1638	
Largest diff. peak and hole	1.175 and –0.998 e Å^{-3}	

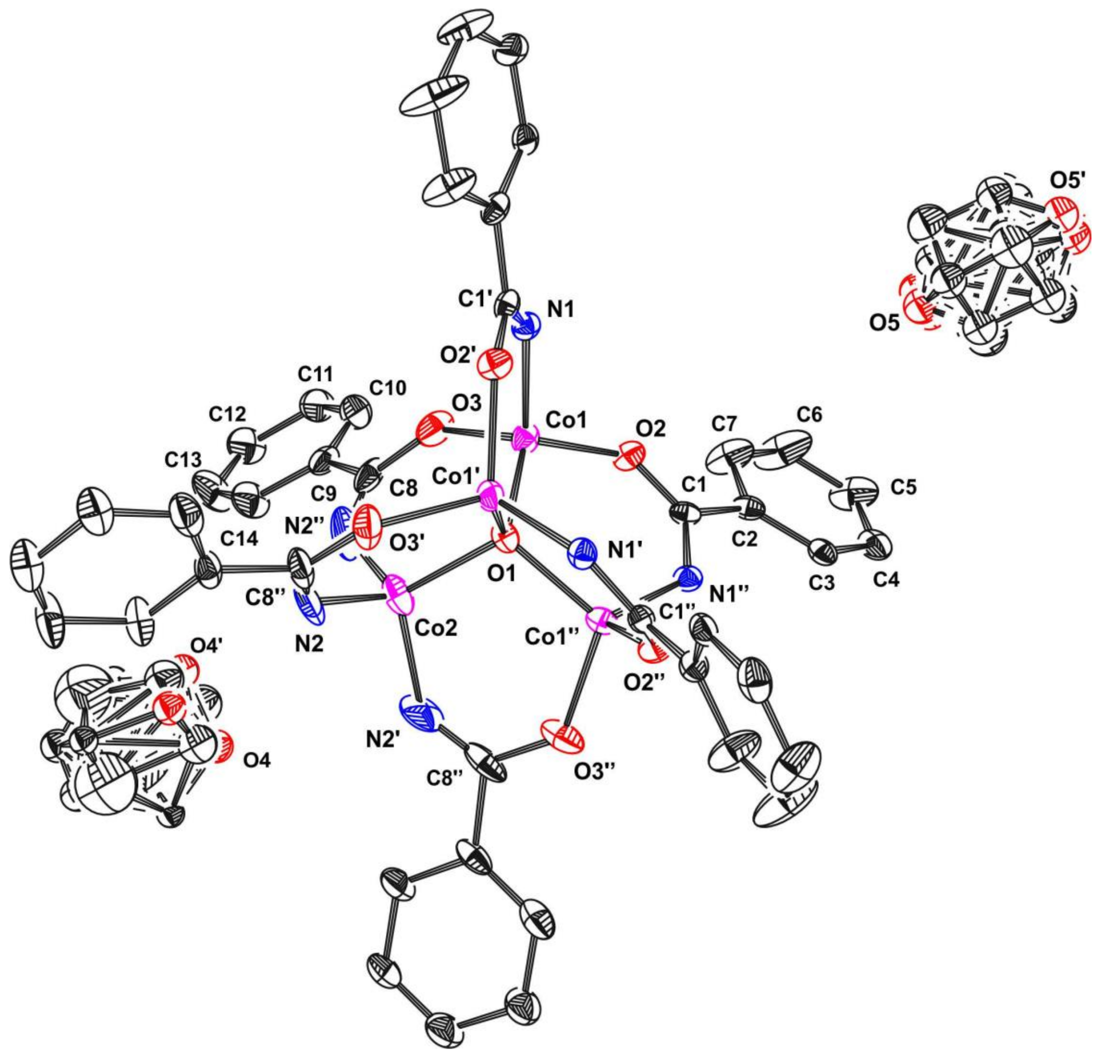

Figure S2. ORTEP diagram of molecular structure of **1-Co** with thermal ellipsoids set at 35% probability; hydrogen atoms have been omitted for clarity. Symmetry transformations used to generate equivalent atoms: (–*x*+*y*+1, –*x*+1, *z*), (–*y*+1, *x*–*y*, *z*)

Table S5. Selected bond lengths [Å] and angles [°] for **1-Co**

Co1 – O1	1.955(2)	Co1 – O1 – Co2	108.50(17)
Co1 – O2	1.941(4)	Co1 – O1 – Co1’	110.42(17)
Co1 – O3	1.996(5)	O1 – Co1 – O2	110.81(15)
Co1 – N1	1.974(5)	O1 – Co1 – O3	112.5(3)
Co2 – O1	1.948(6)	O1 – Co1 – N1	110.60(17)
Co2 – N2	1.956(8)	O1 – Co2 – N2	111.3(2)
C1 – O2	1.267(7)	Co1 –O2 – C1	133.6(4)
C1’ – N1’	1.308(7)	Co1 – O3 – C8	129.5(6)
C8 – O3	1.294(9)	Co1 – N1 – C1’	131.0(4)
C8’’ – N2	1.241(11)	Co2 – N2 – C8’’	134.4(5)

Table S6. Geometry of selected intermolecular hydrogen bonds in **1-Co** (based on Platon)[1]

contact	H···A	D···A	D−H···A	symmetry
N1−H1···O3	2.783	3.635(7)	163.46	*y*, +*x*, −*z*+1/2
N2−H2···O4	2.549	3.342(2)	150.40	−*y*+1, +*x*−*y*, +*z*

Table S7. Crystal data and structure refinement for **1-Zn**

Empirical formula	$C_{42}H_{36}N_6O_7Zn_4$	
Formula weight	998.25 g mol^{-1}	
Temperature	100(2) K	
Wavelength	0.71073 Å	
Crystal system	Trigonal	
Space group	P -3 c 1	
Unit cell dimensions	*a* = 14.540(2) Å	α = 90°
	b = 14.540(2) Å	β = 90°
	c = 25.444(2) Å	γ = 120°
Volume	4658.4(13) Å^3	
Z	4	
Density (calculated)	1.423 Mg/m^3	
Absorption coefficient	2.086 mm^{-1}	
F(000)	2024	
Crystal size	0.18 x 0.10 x 0.04 mm^3	
Theta range for data collection	2.276 to 25.499°	
Index ranges	$-17 \le h \le 13$, $-17 \le k \le 17$, $-23 \le l \le 31$	
Reflections collected	2917	
Independent reflections	2917 [R(int) = 0.0978]	
Completeness to theta = 25.242°	99.9 %	
Refinement method	Full-matrix least-squares on F^2	
Data / restraints / parameters	2917 / 0 / 182	
Goodness-of-fit on F^2	1.020	
Final R indices [I>2σ(I)]	R1 = 0.1397, wR2 = 0.2991	
R indices (all data)	R1 = 0.1546, wR2 = 0.3055	
Largest diff. peak and hole	1.153 and –1.333 e Å^{-3}	

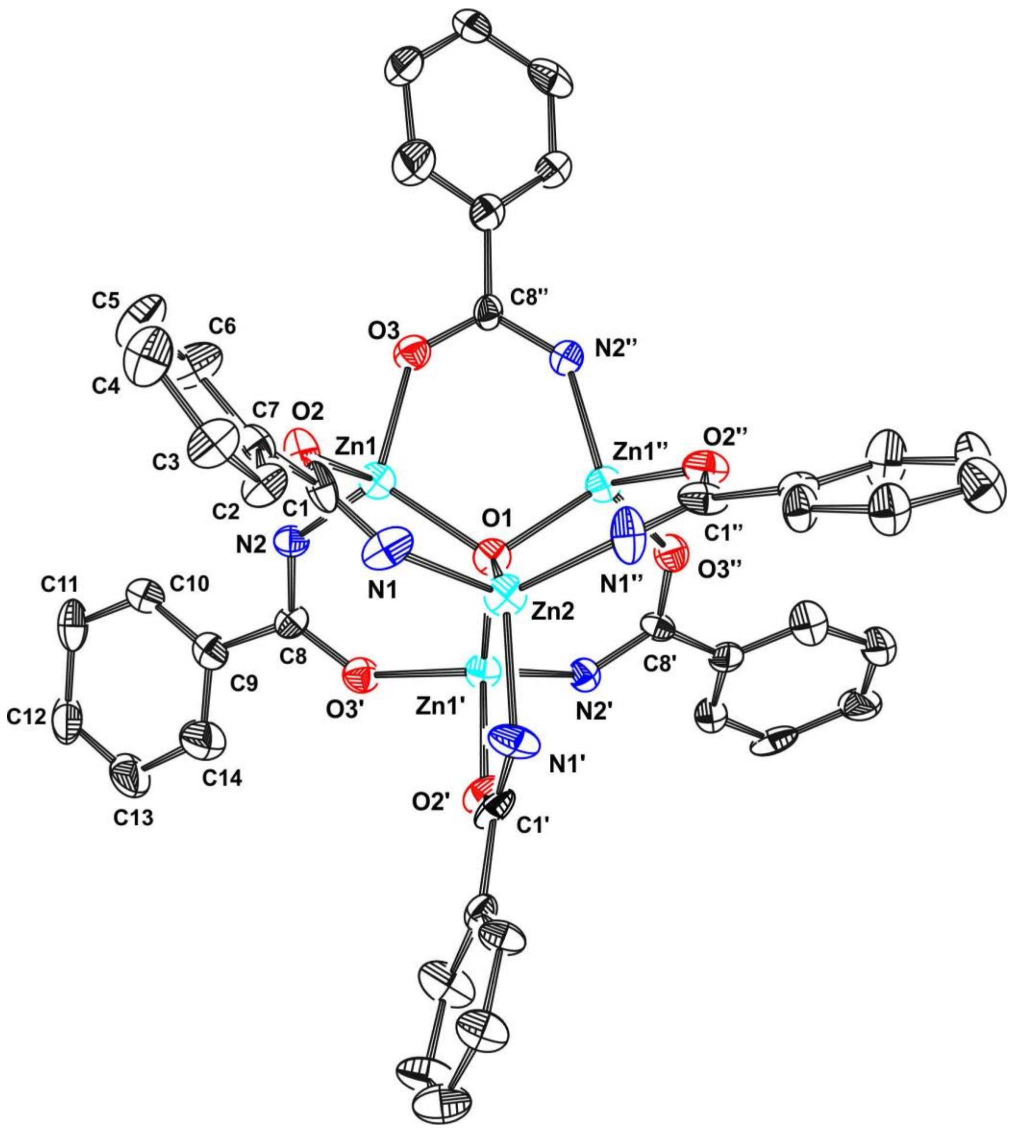

Figure S3. ORTEP diagram of molecular structure of **1-Zn** with thermal ellipsoids set at 35% probability; hydrogen atoms have been omitted for clarity. Symmetry transformations used to generate equivalent atoms: (–*x*+*y*+1, –*x*+1, *z*)   (–*y*+1, *x*–*y*, *z*)

Table S8. Selected bond lengths [Å] and angles [°] for **1-Zn**

Zn1 – O1	1.941(5)	Zn1 – O1 – Zn2	108.1(4)
Zn1 – O2	2.005(10)	Zn1 – O1 – Zn1’	110.8(4)
Zn1 – O3	1.958(10)	O1 – Zn1 – O2	114.7(5)
Zn1 – N2	1.970(11)	O1 – Zn1 – O3	109.5(4)
Zn2 – O1	1.961(14)	O2 – Zn1 – O3	102.0(5)
Zn2 – N1	1.984(14)	O1 – Zn1 – N2	110.6(4)
C1 – O2	1.323(17)	O1 – Zn2 – N1	109.9(4)
C1 – N1	1.22(2)	Zn1 –O2 – C1	127.0(11)
C8 – O3’	1.268(17)	Zn1 – O3 – C8’’	135.5(10)
C8 – N2	1.286(17)	Zn1 – N2 – C8	132.5(11)
		Zn2 – N1 – C1	135.6(10)

Table S9. Geometry of selected intermolecular Hydrogen Bonds: for **1-Co** (based on Platon)[1]

contact	H···A	D···A	D–H···A	symmetry
N2–H2···O2	2.760	3.608(16)	163.0	*y*, +*x*, –*z*+1/2

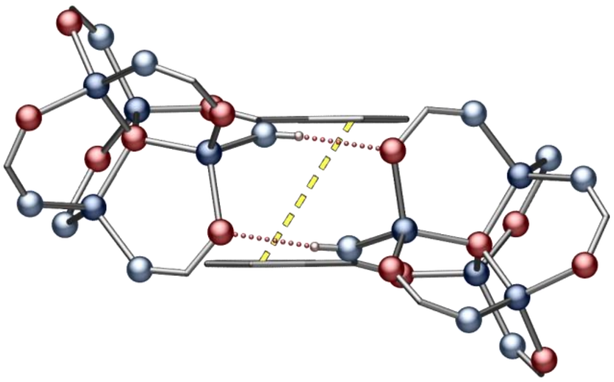

Figure S4. Noncovalent interactions system in the complexes **1-Fe**, **1-Co**, and **1-Zn.**

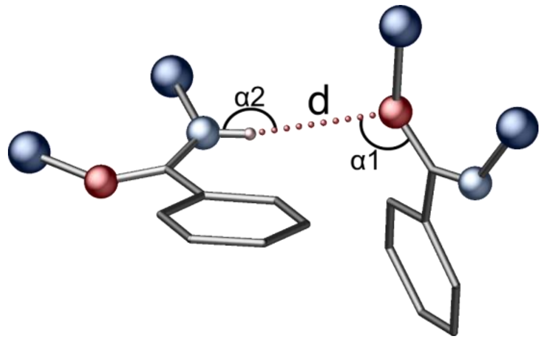

Table S10. Comparison of the geometry of intermolecular hydrogen bonds in **1-Fe**, **1-Co** and **1-Zn**

**M**	**α1 (C-O···H)**	**α2 (N-H···O)**	**d (O···H)**
**Fe**	110.77°	159.79 Å	2.662 Å
**Co**	96.36°	163.46 Å	2.783 Å
**Zn**	94.51°	163.0 Å	2.760 Å

Isomerism

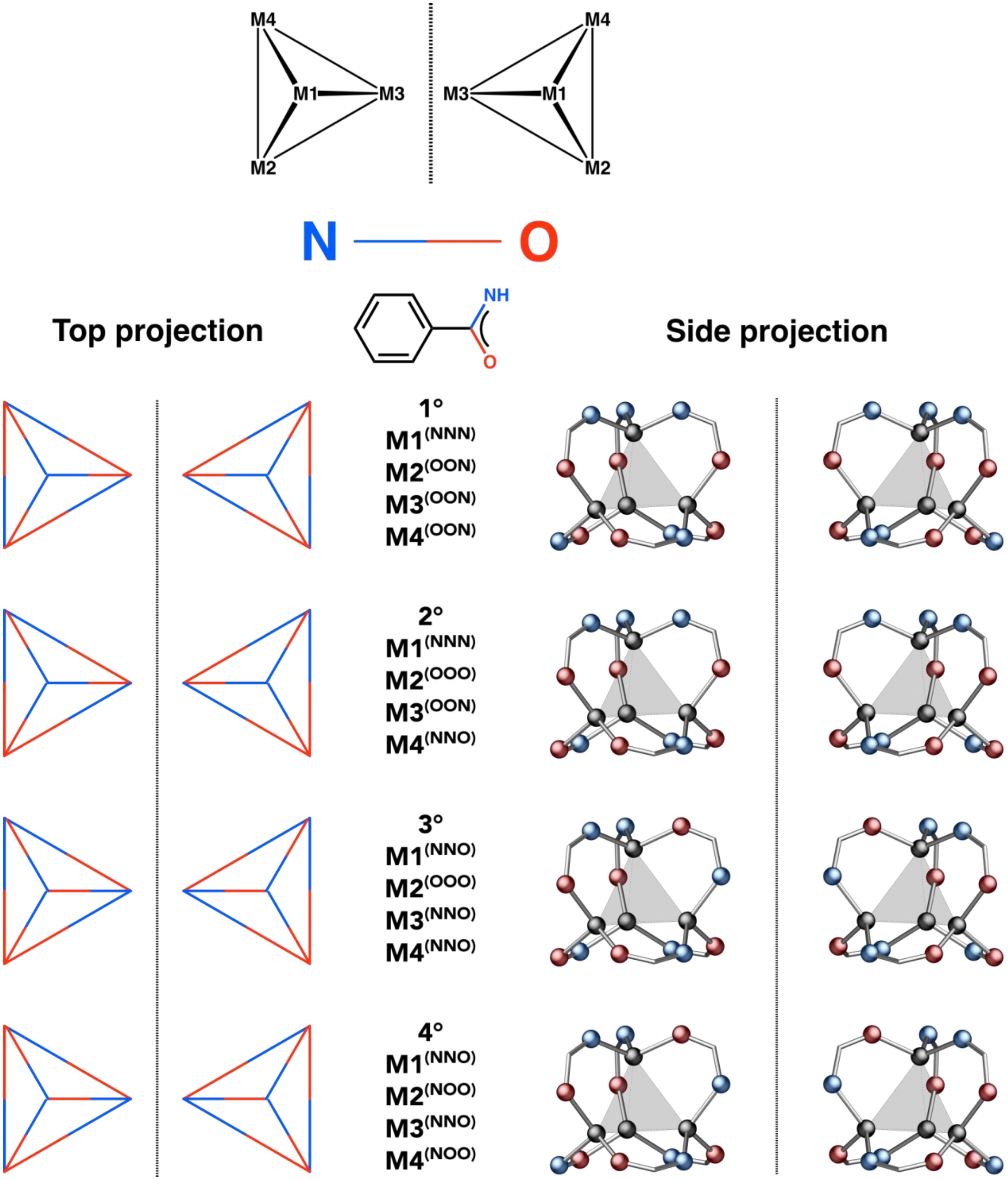

Figure S5. Schematic representation of four possible coordination position isomers of the complexes **1-Fe**, **1-Co** and **1-Zn**, and their enantiomers.

Powder X-Ray Diffraction

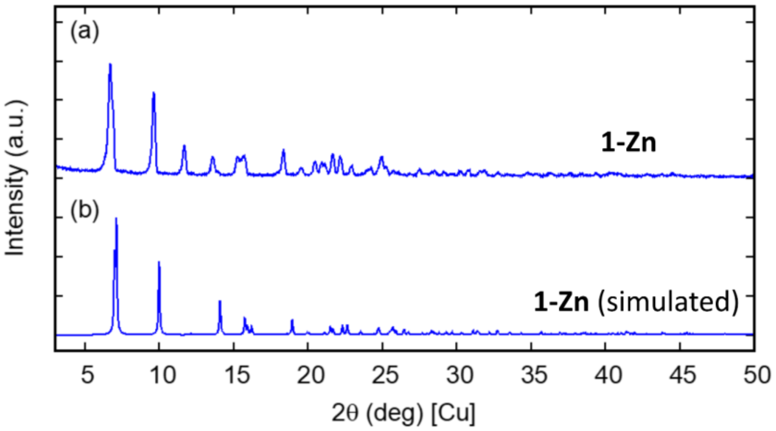

Figure S6. Powder patterns (λ = 1.5406 Å) of **1-Zn**: (a) synthesized bulk phase, (b) simulated from the crystal structure.

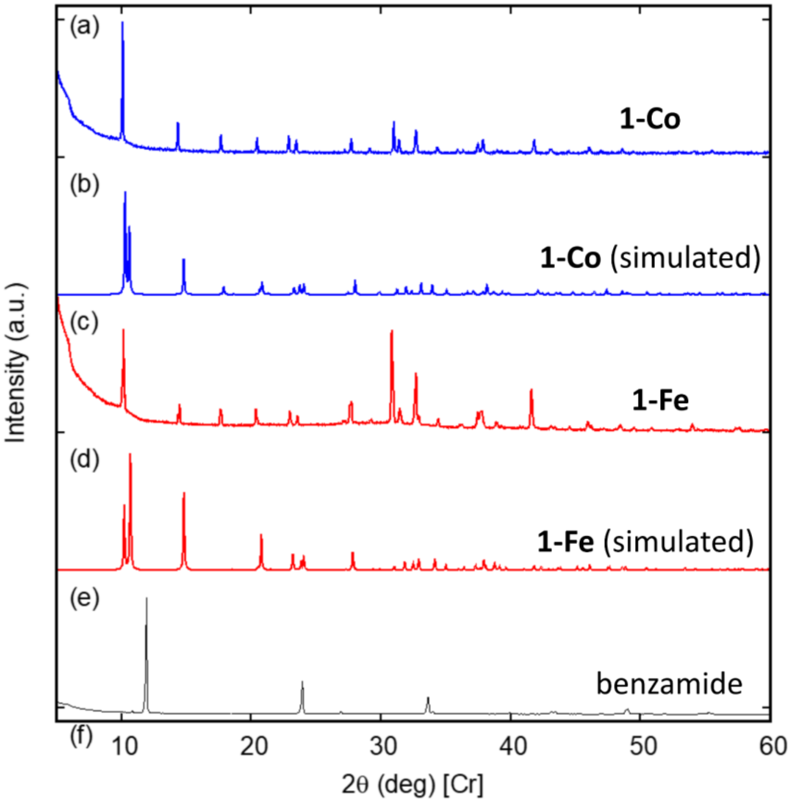

Figure S7. Powder patterns (λ = 2.2936 Å) of (a) bulk phase of **1-Co**, (b) simulated from crystal structure of **1-Co**, (c) bulk phase of **1-Fe**, (d) simulated from crystal structure of **1-Fe**, (e) benzamide.

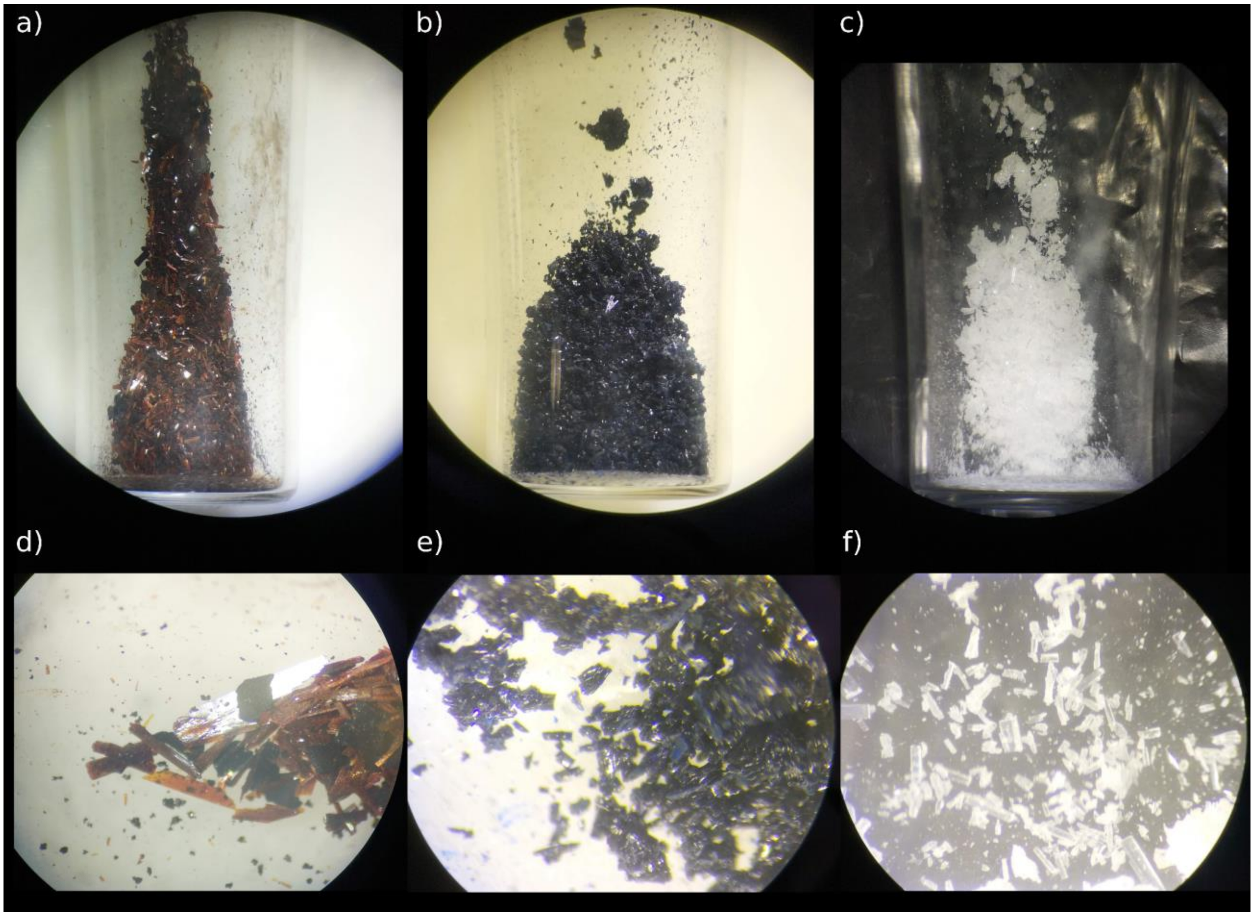

Figure S8. Microscopic images of the bulk samples of **1-Fe** (a,d), **1-Co** (b,e), and **1-Zn** (c,f).

FTIR Spectra

In the ATR-FTIR spectra of **1-Fe**, **1-Co**, and **1-Zn** (Figure S9), the bands observed at 477 (**1-Fe**), 487 (**1-Co**), and 488 cm^{-1} (**1-Zn**), respectively, can be assigned to the asymmetric Fe-O, Co-O and Zn-O vibrations in the [M_4(μ_4-O)] motif. Moreover, the presence of the N-H in-plane bending frequency (**1-Fe**: 1543 cm^{-1}, **1-Co**: 1554 cm^{-1}, **1-Zn**: 1562 cm^{-1}) as well as lack of the NH_2 scissoring band typical of free benzamide (1622 cm^{-1}) confirm the absence of proligand impurities. Strong bands at 1673 and 1449 cm^{-1} in the spectrum of **1-Fe**, at 1673 and 1452 cm^{-1} in **1-Co**, and 1677 and 1454 cm^{-1} in **1-Zn** can be ascribed to C=O and C-N stretching vibrations, respectively. In the light of detailed considerations made earlier for oxido-carboxylate complexes,[2,3] the above observations clearly indicate the presence of μ_2-bonded amidate ligands in the structures of all **1-Fe**, **1-Co** and **1-Zn**.

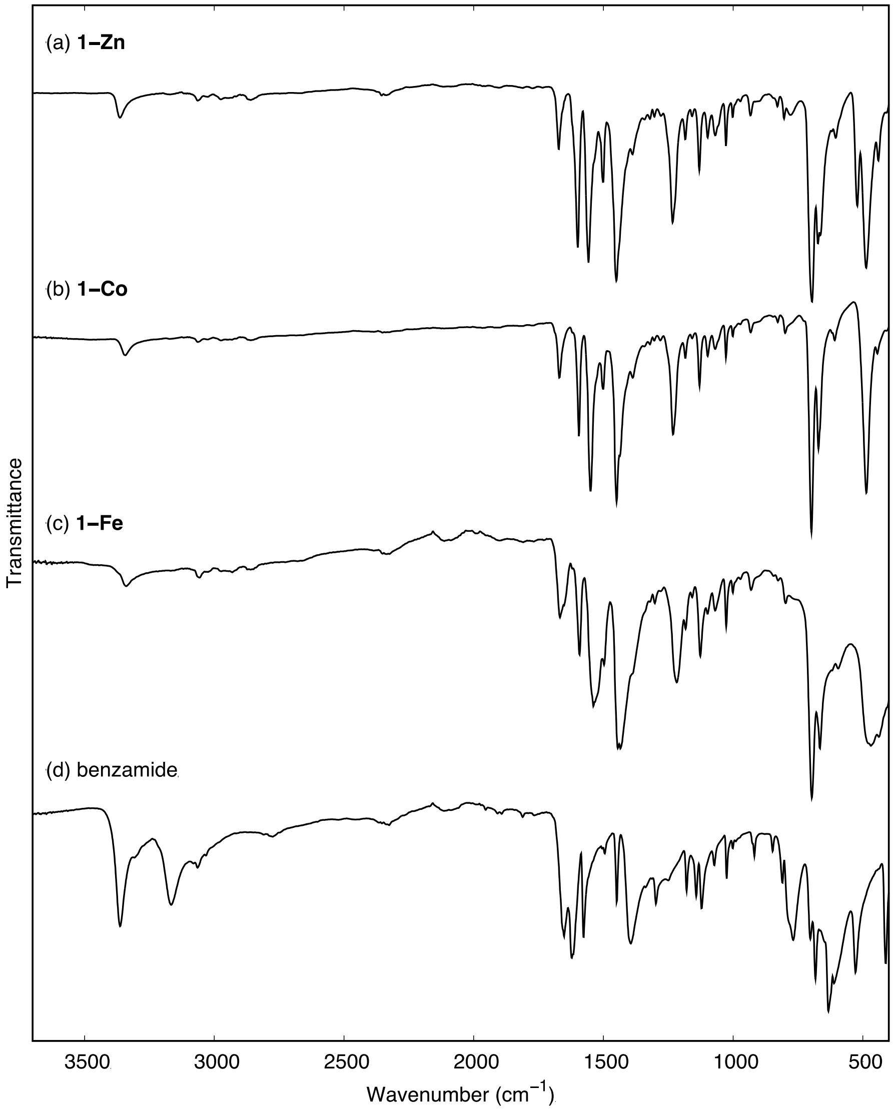

Figure S9. ATR-FTIR spectra of (a) **1-Zn**, (b) **1-Co**, (c) **1-Fe**, and (d) benzamide.

NMR Spectra

^{1}H NMR spectrum of **1-Zn** (Figure S9) shows signals assigned to the aromatic ring and NH group in the ranges 7.3–7.9 ppm, and 6.1–6.4 ppm, respectively. The letter is split into three singlets with 1:3:1 intensity due to the coordination position isomerism (see crystal structure description), which results in various chemical environment of N-bonded protons. Furthermore, the ^{1}H NMR spectrum shows signals of THF and DMF as crystallization media residues, which are encapsulated within the supramolecular framework of **1-Zn** (see crystal structure description). The intensity ratio of signals at 7.90–7.73 ppm (**1-Zn**, 12H), 8.02 (DMF, 1H) and 3.75 (THF, 4H) is 12.00:0.84:3.17, which gives an estimated solvate stoichiometry as 1 complex : 0.84 DMF : 0.79 THF. ^{13}C NMR spectrum of **1-Zn** (Figure S10) shows a set of signals in the range 127–131 ppm from unsubstituted aromatic carbon atoms, and two singlets at 177 and 162 ppm assigned to amide and quaternary aromatic carbon atoms, respectively. Furthermore, there are also signals from THF and DMF residues, which is consistent with other analyses.

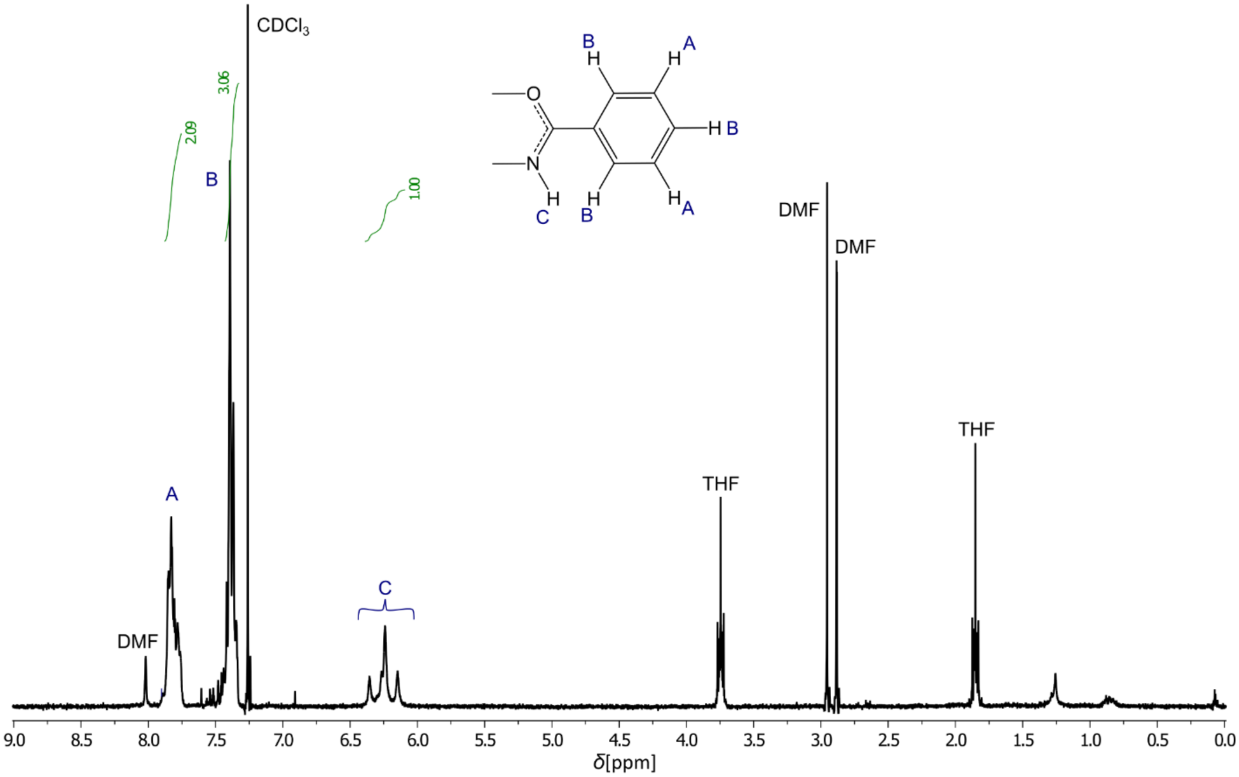

Figure S10. ^{1}H NMR spectrum of **1-Zn**.

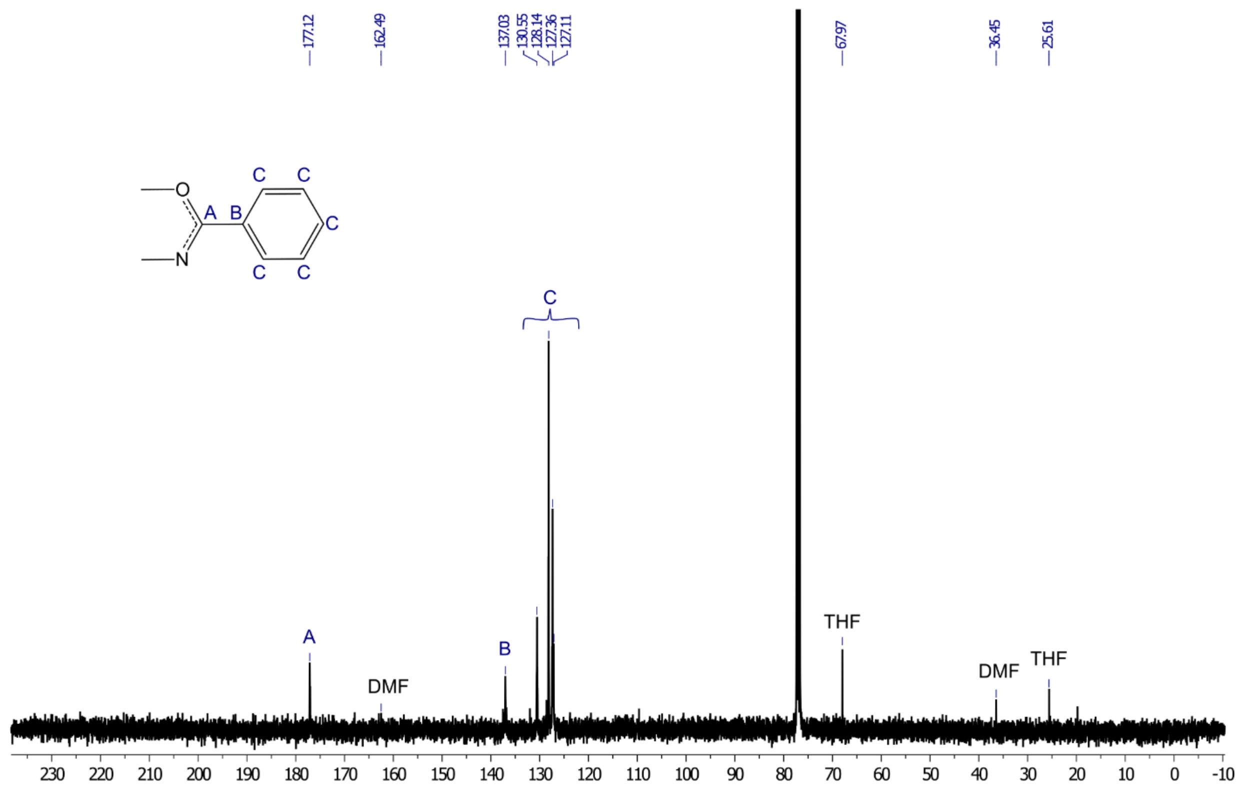

Figure S11. ^{13}C NMR spectrum of **1-Zn**.

Magnetic Studies

The magnetic data of **1-Fe** are shown as $\chi_m T$ vs T plot at 0.1 T and at 1.0 T, and as M_m vs B plot at 2.0 K in Figure 5. At 290 K, the $\chi_m T$ value is 6.30 cm^3 K mol^{-1} that is well below the expected range[4] 13.0–16.3 cm^3 K mol^{-1} of four non-interacting high-spin Fe^{II} centers. This value is also well above the expected value of zero for four low-spin Fe^{II} centers, as a low-spin state is precluded for tetrahedral coordination. Upon cooling, $\chi_m T$ continuously decreases to reach 0.26 cm^3 K mol^{-1} at 2.0 K. At this temperature, the molar magnetization increases up to 1.5 T with a continuously decreasing slope, and almost linearly increases at higher fields reaching 0.32 $N_A \mu_B$ at 5.0 T. Therefore, the data reveal predominantly antiferromagnetically coupled Fe^{II} centers due to the low value of $\chi_m T$ at 290 K, which further continuously decrease upon decreasing values of T and approach zero at the lowest temperatures. In addition, the value of M_m at 5.0 T is also well below the expected range 4.1–4.7 $N_A \mu_B$ of four non-interacting Fe^{II} centers, further indicating predominantly antiferromagnetic exchange interactions. The non-vanishing values of $\chi_m T$ and M_m at 2.0 K are most likely due to the presence of small levels of paramagnetic impurities in the sample batch.

The $\chi_m T$ vs T plot at 0.1 T and 1.0 T of **1-Co** is shown in Figure 6. At 290 K, $\chi_m T$ reaches a value of 6.20 cm^3 K mol^{-1}. This is well below the anticipated range[4] 9.2–13.5 cm^3 K mol^{-1} for four non-interacting tetrahedrally coordinated Co^{II} centers. With decreasing temperatures, $\chi_m T$ continuously decreases, reaching 0.10 cm^3 K mol^{-1} at 7.5 K, and subsequently proceeds to decrease with a distinctly smaller slope to 0.03 cm^3 K mol^{-1} at 2.0 K. The molar magnetization M_m at 2.0 K (see inset, Figure 6) is almost linear up to 5.0 T, and reaches a value less than 0.1 $N_A \mu_B$ at 5.0 T (about 0.08 $N_A \mu_B$). The data, thus, identify predominantly antiferromagnetic exchange interactions between the four Co^{II} centers. This is reflected by the shape of the $\chi_m T$ vs T plot, by the rather low value of $\chi_m T$ at 290 K, and the almost vanishing values of $\chi_m T$ and M_m at 2.0 K representing an effective total spin value of $S_{total} = 0$.

References

(1) Spek, A. L. Structure Validation in Chemical Crystallography. *Acta Crystallogr. Sect. D Biol. Crystallogr.* **2009**, *65* (2), 148–155 DOI: 10.1107/S090744490804362X.

(2) Johnson, M. K.; Powell, D. B.; Cannon, R. D. Vibrational Spectra of Carboxylato Complexes-II. Some Oxo-Tetranuclear Complexes. *Spectrochim. Acta Part A Mol. Spectrosc.* **1982**, *38* (2), 125–131 DOI: 10.1016/0584-8539(82)80187-6.

(3) Clegg, W.; Harbron, D. R.; Homan, C. D.; Hunt, P. A.; Little, I. R.; Straughan, B. P. Crystal Structures of Three Basic Zinc Carboxylates Together with Infrared and FAB Mass Spectrometry Studies in Solution. *Inorganica Chim. Acta* **1991**, *186* (1), 51–60 DOI: 10.1016/S0020-1693(00)87930-X.

(4) Lueken, H. *Magnetochemie*; Teubner Studienbücher Chemie; Vieweg+Teubner Verlag: Wiesbaden, 1999.
